# Pharmacologic inhibition of phospholipase C in the brain attenuates early memory formation in the honeybee (*Apis mellifera* L.)

**DOI:** 10.1242/bio.028191

**Published:** 2018-01-15

**Authors:** Shota Suenami, Shiori Iino, Takeo Kubo

**Affiliations:** 1 Bioproduction Research Institute, National Institute of Advanced Industrial Science and Technology, Ibaraki 305-8566, Japan; 2 Department of Biological Sciences, Graduate School of Science, The University of Tokyo, Bunkyo-ku, Tokyo 113-0033, Japan

**Keywords:** Honeybee, Learning and memory, Phospholipase C, Behavioral pharmacology, Brain

## Abstract

Although the molecular mechanisms involved in learning and memory in insects have been studied intensively, the intracellular signaling mechanisms involved in early memory formation are not fully understood. We previously demonstrated that *phospholipase C epsilon* (*PLCe*), whose product is involved in calcium signaling, is almost selectively expressed in the mushroom bodies, a brain structure important for learning and memory in the honeybee. Here, we pharmacologically examined the role of phospholipase C (PLC) in learning and memory in the honeybee. First, we identified four genes for PLC subtypes in the honeybee genome database. Quantitative reverse transcription-polymerase chain reaction revealed that, among these four genes, three, including *PLCe*, were expressed higher in the brain than in sensory organs in worker honeybees, suggesting their main roles in the brain. Edelfosine and neomycin, pan-PLC inhibitors, significantly decreased PLC activities in homogenates of the brain tissues. These drugs injected into the head of foragers significantly attenuated memory acquisition in comparison with the control groups, whereas memory retention was not affected. These findings suggest that PLC in the brain is involved in early memory formation in the honeybee. To our knowledge, this is the first report of a role for PLC in learning and memory in an insect.

## Abstract (precis)

**Summary:** Intracellular signaling involved in early memory formation in insects is not fully understood. Here, we pharmacologically elucidated the role of phospholipase C in learning and memory in the honeybee.

## INTRODUCTION

An animal's ability to learn and memorize information through experience facilitates its behavioral adaptation under changing circumstances. Although the molecular mechanisms underlying learning and memory have been intensively studied using various animals (Elgersma et al., 2004; Davis, 2011; Menzel, 2012), the depth of understanding of these mechanisms differs depending on the animal species. Thus, the similarities and differences of the molecular mechanisms involved in learning and memory among animal species are not fully understood.

The honeybee (*Apis mellifera* L.) is a social insect, and females differentiate into a reproductive caste (‘queen’) or sterile caste (‘worker’). Workers show age-polyethism, and young workers feed their broods while older ones forage outside the hives. Foragers learn and memorize the location of their nest and food sources, and repeatedly visit the same food source (Chittka et al., 1999). If a forager succeeds in finding a good food source, the bee communicates the location and quality of the source to its nestmates using dance (Winston, 1986). Thus, learning and memory abilities are important in the honeybee.

Although extensive studies of the molecular mechanisms underlying honeybee learning and memory have been performed, the molecules contributing to the intracellular signaling involved in early memory formation in the honeybee remain unknown, and may differ from those in other animals and insects. For example, pharmacologic inhibition of adenylate cyclase, whose subtype is important in learning and memory in the fruit fly *Drosophila melanogaster* (McGuire et al., 2005; Trannoy et al., 2011), does not influence short-term memory in the honeybee (Matsumoto et al., 2014).

We previously demonstrated that the expression of some genes involved in calcium signaling, such as *inositol 1, 4, 5-trisphosphate receptor* (*IP_3_R*), *calcium/calmodulin-dependent protein kinase II* (*CaMKII*), *protein kinase C* (*PKC*), *type I-IP_3_ 5-phosphatase*, *ryanodine receptor*, *reticulocalbin*, and *phospholipase C epsilon* (*PLCe*), are enriched in the honeybee mushroom bodies (MBs), which is a higher brain center in insects (Kamikouchi et al., 1998, 2000; Takeuchi et al., 2002; Uno et al., 2013; Suenami et al., 2016). Although there are several phospholipase C (PLC) subtypes that are stimulated by different mechanisms, all PLC subtypes trigger calcium signaling by degrading phosphatidylinositol into IP_3_ and diacylglycerol (Smrcka et al., 2012). IP_3_ binds and opens IP_3_R, which releases calcium ions from intracellular calcium stores. Calcium activates CaMKII and PKC in combination with calmodulin and diacylglycerol, respectively (Ghosh and Greenberg, 1995; Smrcka et al., 2012; Dusaban and Brown, 2015). Considering that mammals utilize calcium signaling in learning and memory (Elgersma et al., 2004; Giese and Mizuno, 2013) and that the MBs are known to be involved in honeybee learning and memory (Lozano et al., 2001; Müßig et al., 2010), the enriched expression of genes involved in calcium signaling in the honeybee MBs suggests that this type of signaling is important in learning and memory in the honeybee.

In the present study, we focused on PLC, a highly important enzyme in calcium signaling. Evaluation of the role of PLC in learning and memory in the honeybee using pharmacologic inhibitors revealed that PLC inhibition attenuates memory acquisition. This is the first study, to our knowledge, to identify an intracellular signaling molecule involved in early memory formation in the honeybee.

## RESULTS

### Identification of PLC subtypes encoded in the honeybee genome

Previous studies of gene expression profiles in the honeybee brain identified several PLC subtypes (Sen Sarma et al., 2009; Suenami et al., 2016), although the complete list of PLC subtypes was not examined in these studies. As the first step in the present study, we searched for genes encoding PLC subtypes other than *PLCe* in the honeybee genome to gain insight into signaling involving PLC subtypes in the honeybee.

**Fig. 1.**
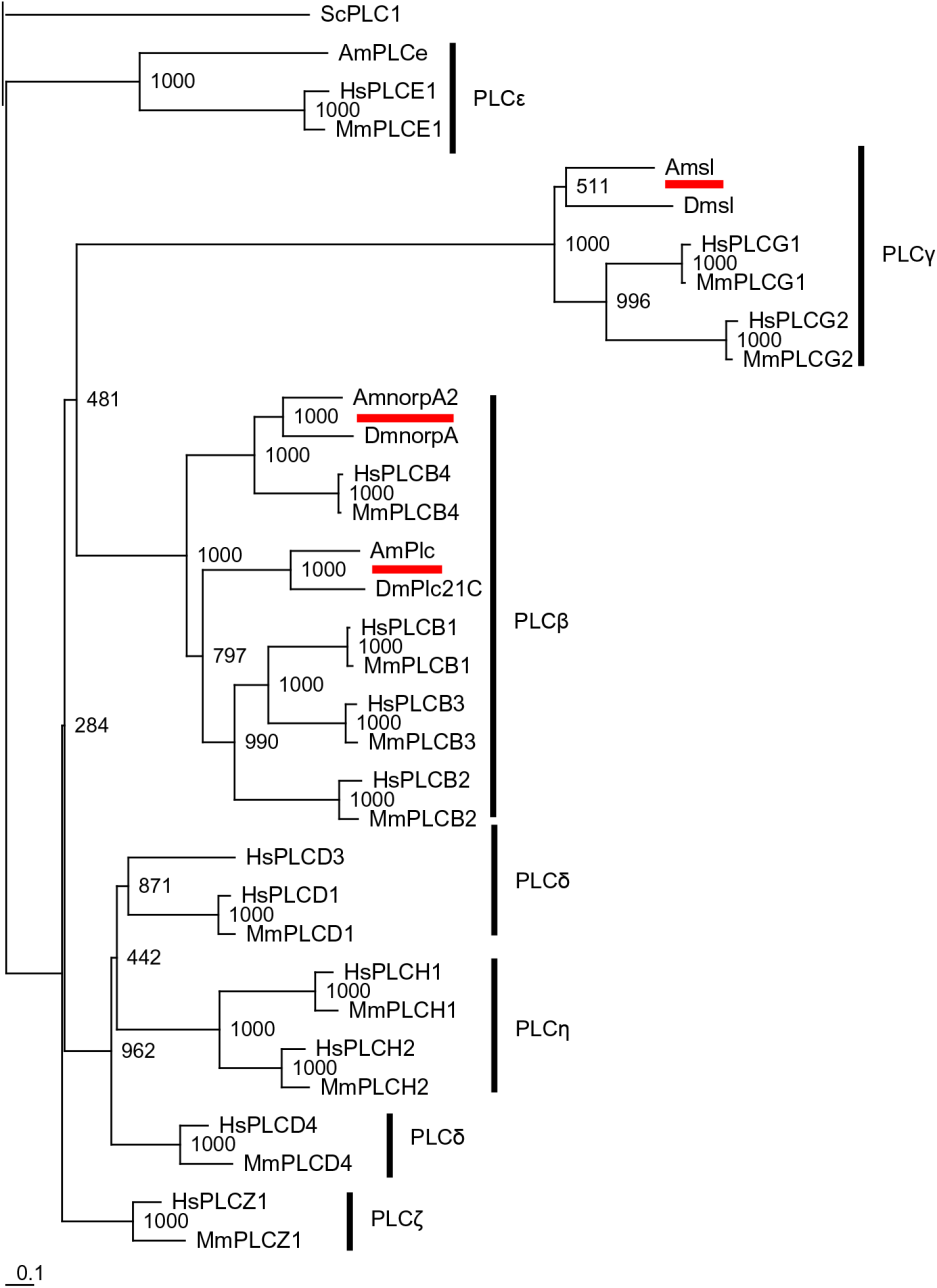
**Phylogenetic analysis of AmPlc, AmnorpA2, and Amsl.** PLC proteins from the honeybee, fruit fly, mouse, human, and yeast were analyzed. AmPlc, AmnorpA2, and Amsl are underlined. Yeast PLC1 was included as an outgroup. Numbers at branches represent bootstrap values in 1000 trials. Scale bar is shown at the bottom left (0.1 amino acid substitutions per site). Abbreviations of species are shown before identities of PLC subtypes, as follows: Am, *Apis mellifera*; Dm, *Drosophila melanogaster*; Mm, *Mus musculus*; Hs, *Homo sapiens*; Sc, *Saccharomyces cerevisiae*. PLCβ, PLCγ, PLCδ, PLCε, PLCη, and PLCζ are represented as PLCB, PLCG, PLCD, PLCE, PLCH, and PLCZ, respectively. Plc, phospholipase C; Plc21C, phospholipase C at 21C; norpA, no receptor potential A; norpA2, no receptor potential A2; sl, small wing.

A blastp search for PLC subtypes using the following queries in *D*. *melanogaster*: phospholipase C at 21C (Plc21C), no receptor potential A (norpA), and small wing (sl), identified *A*. *mellifera* phospholipase C (AmPlc), no receptor potential A2 (AmnorpA2), and small wing (Amsl) as the most highly related proteins, respectively, in the NCBI honeybee genome database. We then aligned AmPlc, AmnorpA2, Amsl, and PLCe with PLC subtypes in the fruit fly, mouse (*Mus musculus*), human (*Homo sapiens*), and yeast (*Saccharomyces cerevisiae*), and constructed a phylogenetic tree (Fig. 1). AmPlc, AmnorpA2, and Amsl were placed in the same clade as Plc21C, norpA, and sl, respectively, consistent with the blastp search. While the amino acid identity between AmPlc and Plc21C was high (58.6% of all amino acids, Table 1A), mammalian PLCβ1-3 was not very similar to AmPlc, although they were placed in the same clade (37.5% of all residues, at most). AmnorpA2 had high identity to norpA, human and mouse PLCβ4 (63.1%, 52.5%, and 53.2% of total amino acids, respectively; Table 1B). Amsl showed moderate identity to sl and mammalian PLCγ (39.1% to 49.9% of amino acids; Table 1C) compared with those of AmPlc and AmnorpA2.

**Table 1.**
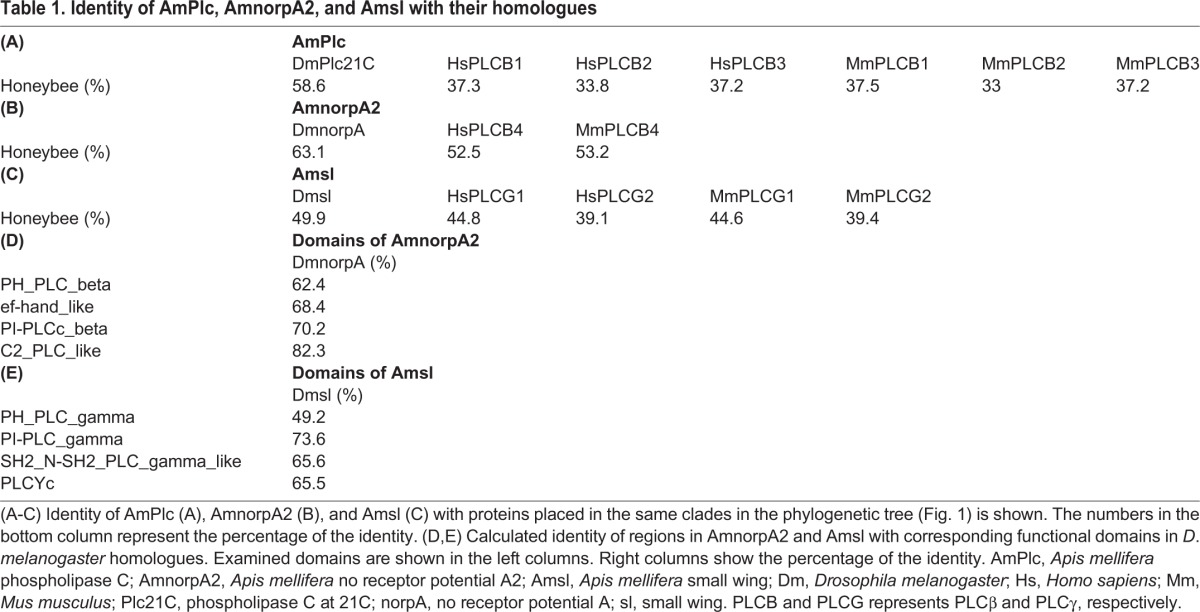
**Identity of AmPlc, AmnorpA2, and Amsl with their homologues**

**Fig. 2.**
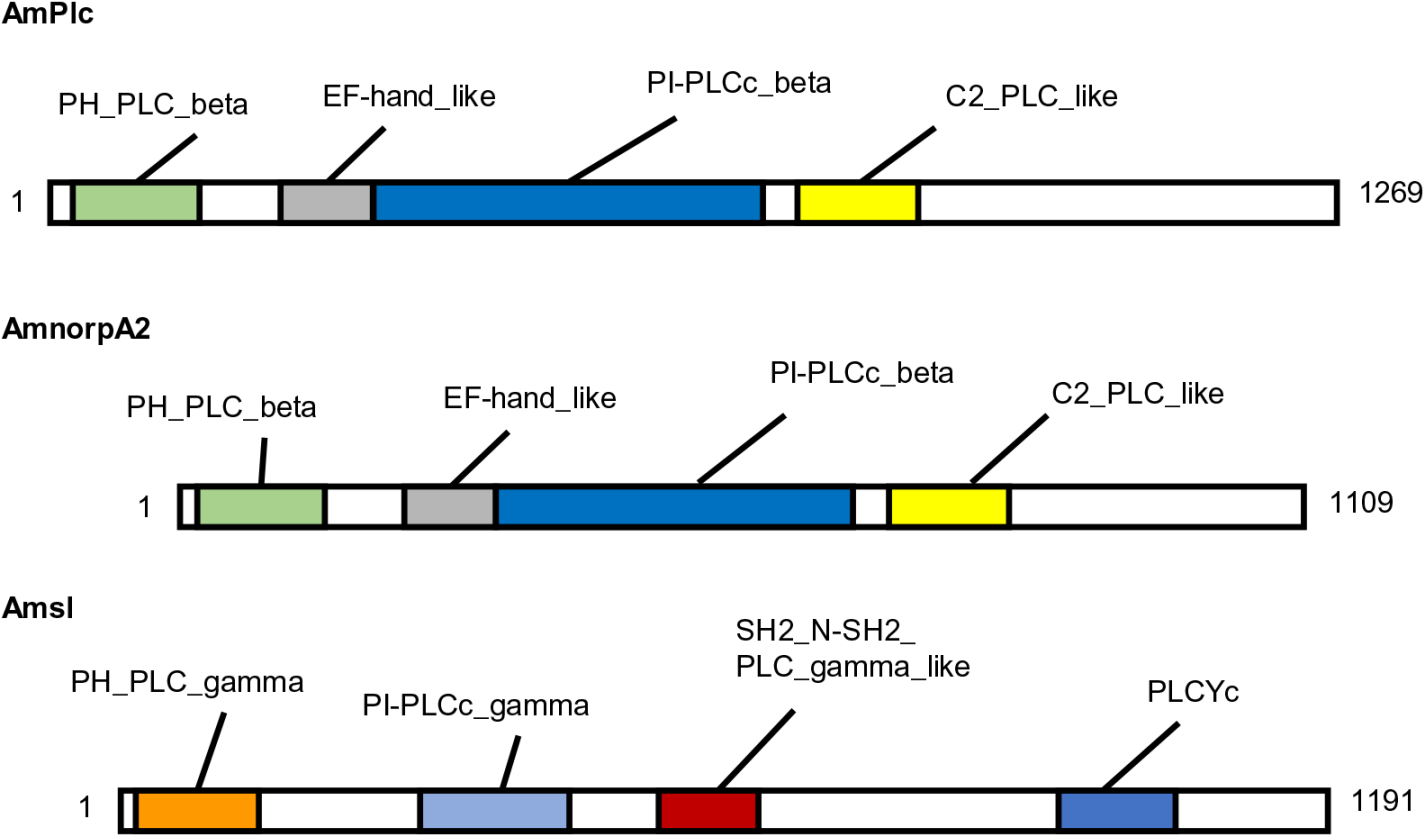
**Schematic illustration of protein structures of AmPlc, AmnorpA2, and Amsl.** The numbers and color boxes represent positions of amino acids and conserved domains, respectively. Domains of AmPlc were obtained from the NCBI database. Domains of AmnorpA2 and Amsl were determined through alignment with their homologues in the fruit fly, mouse, and human. AmPlc, *Apis mellifera* phospholipase C; AmnorpA2, *Apis mellifera* no receptor potential A2; Amsl, *Apis mellifera* small wing.

PLC subtypes in *D*. *melanogaster* are functionally classified based on some conserved domains in the NCBI. For example, Plc21C and norpA were classified as ‘PH_PLC_beta and PI-PLCc_beta domain-containing proteins’, because these proteins had PH_PLC_beta, EF-hand_like, PI-PLCc_beta, and C2_PLC_like domains, while sl was classified as a ‘PH_PLC_gamma and PI-PLCc_gamma domain-containing protein’ based on PH_PLC_gamma, PI-PLCc_gamma, SH2_N-SH2_PLC_gamma, and PLCYc domains. As conserved domains suggest important function, we next examined whether the honeybee PLC subtypes have similar functions as their homologues in *D*. *melanogaster* based on conservation of these domains. Domains of AmPlc were already annotated and this protein was classified as a ‘PH_PLC_beta and PI-PLCc_beta domain-containing protein’ based on the domains in the NCBI shown in Fig. 2. Thus, we did not analyze this protein. To identify the similarity of domains between AmnorpA2 and norpA, AmnorpA2 was aligned with homologues in the fruit fly, mouse, and human, and the regions in AmnorpA2 that corresponded to conserved domains in norpA were determined (Fig. 2). The identity of these regions was high between the honeybee and fruit fly (62.4% to 82.3% of amino acid residues; Table 1D), suggesting that these functional domains are conserved in AmnorpA2. When regions in Amsl corresponding to conserved domains in the fruit fly homologue were determined (Fig. 2), these regions exhibited overall high identity to sl (49.2% to 73.6% of residues; Table 1E) and were considered to be conserved between Amsl and sl.

A blastp search using mouse PLCδ, PLCη, and PLCζ as queries identified AmnorpA2 or Amsl as the most similar proteins, suggesting that the honeybee does not have PLCδ, PLCη, or PLCζ. These results suggest that the honeybee shares PLC subtypes with the fruit fly, except for PLCe.

### Analysis of expression of PLC subtype genes in honeybee tissues involved in associative learning

To identify the candidate PLC subtypes involved in honeybee learning and memory, we analyzed the expression levels of four PLC subtype genes in the brain and sensory organs of the honeybee using quantitative reverse-transcription polymerase chain reaction (qRT-PCR). Insect brains have some characteristic structures in addition to the MBs, such as the antennal lobe (AL), optic lobe (OL), and suboesophageal ganglion (SOG), which are the primary sensory centers of olfaction, vision, and gustation, respectively. Here, we separately analyzed the MBs and other brain regions including AL, OL, and SOG. Some sensory organs (retinae, proboscises, and antennae) were also used because they receive visual (retinae), gustatory (proboscises), and olfactory (antennae) stimuli, which are involved in learning and memory.

First, we analyzed *Ribosomal protein L32* (*RpL32*), *elongation factor 1 alpha-F2* (*EF1α-F2*), and *actin related protein 1* (*Arp1*) to determine the optimal reference gene. Because we analyzed *PLCe* and the other PLC genes in different sets of RNA samples, quantification of *RpL32*, *EF1α-F2*, and *Arp1* was performed for each sample set. Expression levels for all genes differed significantly among tissues (Fig. S1). While *EF1α-F2* and *Arp1* were scarcely or not detected in some retina samples, *RpL32* was detected in all tissues (Fig. S1A,C and E). In addition, *RpL32* exhibited the smallest change in expression among tissues other than the retinae: *RpL32* expression changed approximately 2.6-fold at most, while *EF1α-F2* and *Arp1* expression changed approximately 3.1-fold and 2.9-fold at most, respectively (Fig. S1B,D and F). Therefore, we used *RpL32* as a reference gene for our qRT-PCR experiments.

**Fig. 3.**
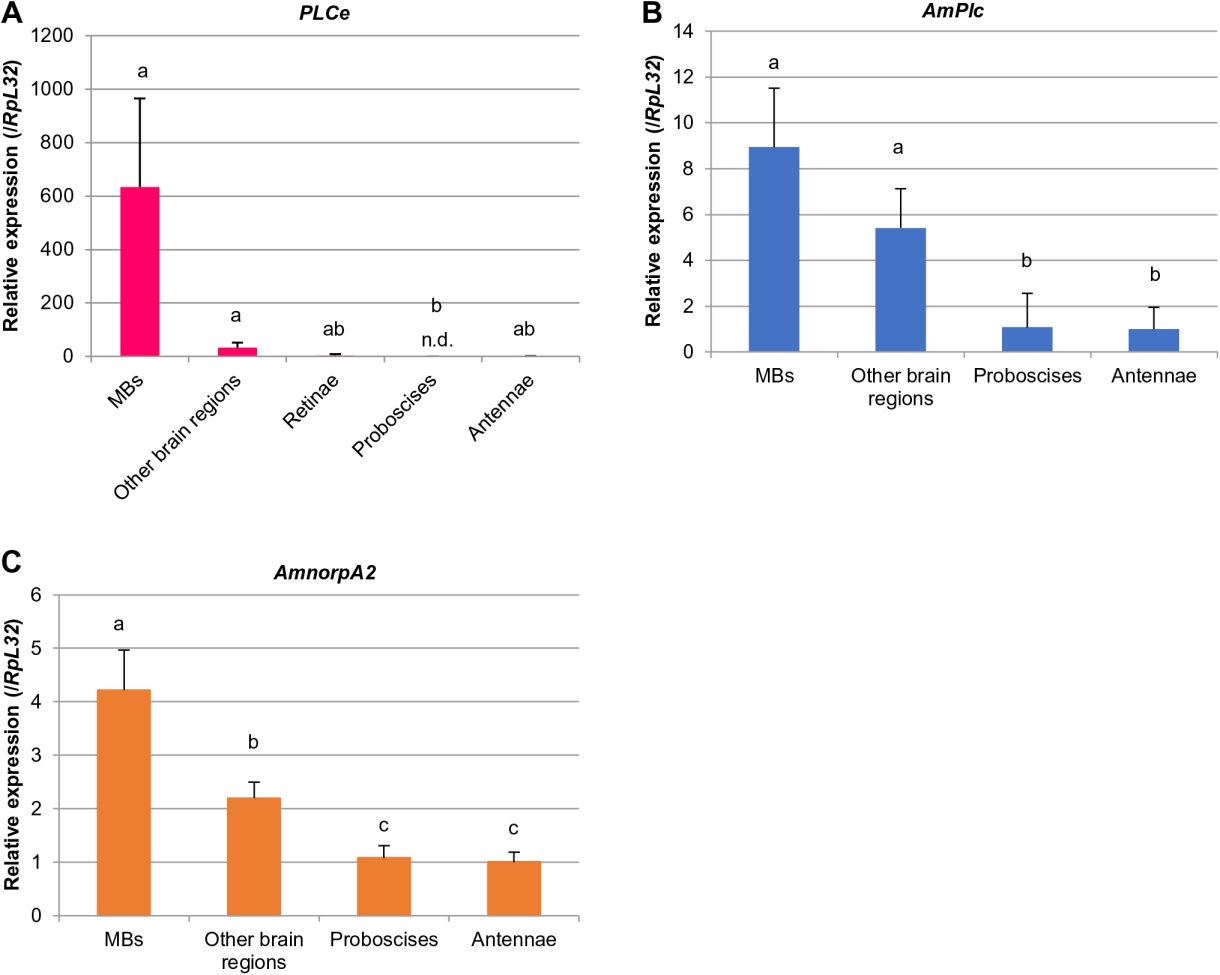
**qRT-PCR analysis of *PLCe*, *AmPlc*, *AmnorpA2,* and *Amsl* in tissues involved in learning and memory in the honeybee.** Expression levels of *PLCe* (A), *AmPlc* (B), *AmnorpA2* (C) in the MBs, other brain regions, retinae (for only *PLCe*), proboscises, and antennae were normalized by that of *RpL32*. Relative expression in the antennae is shown as one. Bars and lines indicate mean of expression levels and s.d. of five lots, respectively. Each lot contained two workers. The experiments shown here were performed once. A significant difference among tissues was detected in the analysis of each gene shown (*P*<0.000 5 for *PLCe* and *P*<0.005 for *AmPlc* and *AmnorpA2*, Kruskal–Wallis test). Different letters on the bars represent a significant difference (*P*<0.05, Steel-Dwass test). Amounts of *PLCe* in four retinae and one antennae, and *AmPlc* in three proboscises and two antennae were below the detection limit and analyzed as zero. Manipulation of these samples was not considered problematic because *RpL32* was detected in all tissues. *Amsl* was not detected in any tissues analyzed (not shown). n.d., not detected. *AmPlc*, *Apis mellifera phospholipase C*; *AmnorpA2*, *Apis mellifera no receptor potential A2*; *Amsl*, *Apis mellifera small wing*; *RpL32*, *Ribosomal protein L32*.

The relative expression of *PLCe*, *AmPlc*, and *AmnorpA2*, which was normalized to that of *RpL32*, was higher in the MBs than in the other brain regions by approximately 19-, 1.7-, and 1.9-fold, respectively (Fig. 3). Sensory organs had lower expression levels than brain tissues (Fig. 3). To be sure, we also analyzed the relative expression of *PLCe* and other *PLC*s in tissues other than the retinae normalized with the expression of *EF1α-F2* and *Arp1*, respectively (Fig. S2). As expected, essentially the same results were obtained when we used *EF1α-F2* and *Arp1* as reference genes instead of *RpL32*: expression of *PLCe* was almost specific to MBs among the tissues, and expression of *AmPlc* and *AmnorpA2* was also higher in the MBs than in the other tissues. No significant expression of *Amsl* was detected in any tissues (not shown).

These findings suggest that PLC signaling is enhanced in the honeybee MBs, and raise the possibility that three PLC subtypes are involved in learning and memory in the honeybee brain. The higher expression of *PLCe* in the MBs than in the other brain regions in the present study is consistent with our previous report (Suenami et al., 2016), although the difference was not significant in the present study, possibly due to the small sample numbers relative to the number of tissues analyzed.

### Inhibitory effects of edelfosine and neomycin on PLC activity in honeybee brain homogenates

Edelfosine and neomycin are widely used to inhibit PLC in mammals and insects (Lipsky and Lietman, 1982; Walz et al., 2000; Koganezawa and Shimada, 2002; Wenzel et al., 2002; Aravamudan and Broadie, 2003; Wong et al., 2007), and we planned to use these drugs to examine PLC involvement in honeybee learning and memory. The effects of these drugs on PLC activity in honeybee brain tissues, however, has not yet been biochemically investigated. Thus, we first tested whether edelfosine and neomycin inhibit honeybee PLC activity using homogenates of the honeybee MBs and other brain regions before performing the behavioral experiments.

**Fig. 4.**
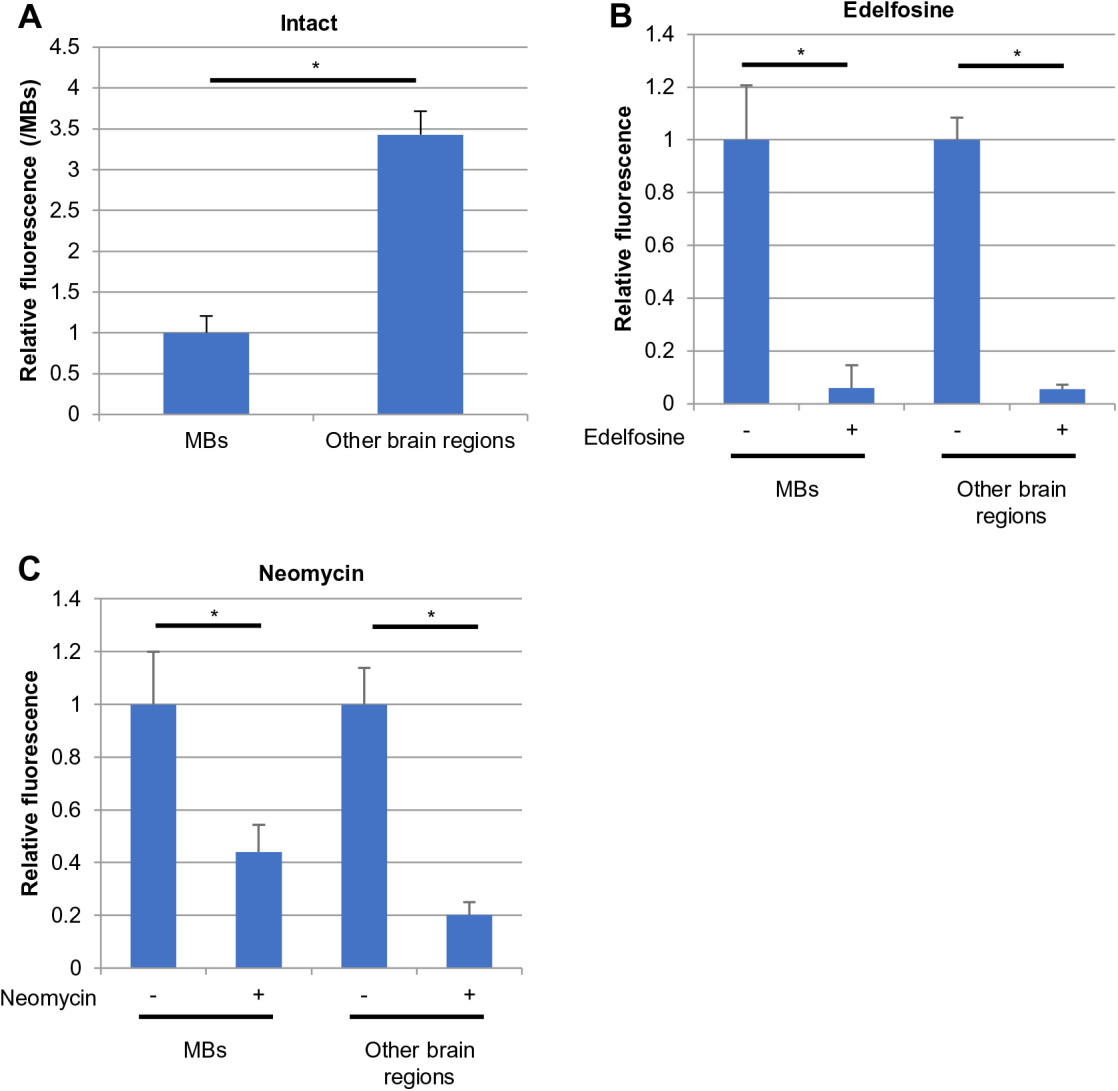
**Inhibitory effects of edelfosine and neomycin on PLC activity in honeybee brain homogenates.** Fluorescence detected in reaction mixtures containing substrate and homogenates constructed from the MBs and other brain regions (A), reaction mixture additionally containing edelfosine (B) or neomycin (C) are shown. In A, homogenates contained the same amounts of proteins, and fluorescence was normalized with that in the MBs. *P<0.005 (Mann–Whitney's U test). In B and C, fluorescence in mixture without drugs is shown as one for each tissue. *P<0.05 (Wilcoxon signed-ranks test). Mean and s.d. of six lots are shown. Each lot contained two workers. Detection of fluorescence was performed with technical triplicates (i.e. three times with the same set of six lots of samples), and the statistical mean value for each fluorescence was determined and analyzed. s.d., standard deviation.

We examined whether PLC activity in each homogenate could be detected using PLCglow, a fluorogenic substrate (Huang et al., 2011). Fluorescence was more than 4.2-fold higher in reaction mixes containing both homogenate and substrate than in control mixes that contained either homogenate or substrate (data not shown), suggesting that the increased fluorescence detected in the reaction mixes reflected PLC enzymatic activity. When an increase in the fluorescence was calculated between the reaction mix and control mixes, the MB homogenate showed less of an increase in the fluorescence than the other brain regions (Fig. 4A). This was unexpected, considering the expression patterns of PLC subtype genes in these tissues. Based on this result, we determined reaction conditions in which fluorescence increased with the reaction time and concentration of the proteins using the homogenate from the other brain regions.

Addition of 1.0 mmol/l edelfosine or 0.55 mmol/l neomycin into the same homogenates significantly altered the fluorescence in both tissue homogenates. Edelfosine decreased fluorescence to approximately 6.0% (MBs) and 5.4% (other brain regions) of the fluorescence detected in mixes without edelfosine (Fig. 4B). Neomycin also reduced fluorescence in the reaction mixes to approximately 44% (MBs) and 20% (other brain regions) of the fluorescence in samples without the drug (Fig. 4C). As these drugs share only PLC as a common target (according to online catalogues for Santa Cruz Biotechnology, Inc.), a decrease in fluorescence was considered to reflect a decrease in the PLC activity. Thus, these results suggested that both edelfosine and neomycin inhibit PLC activity in honeybee brain tissues, at least *in vitro*.

### Analysis of the effects of PLC inhibitors on olfactory associative learning and memory in the honeybee

**Fig. 5.**
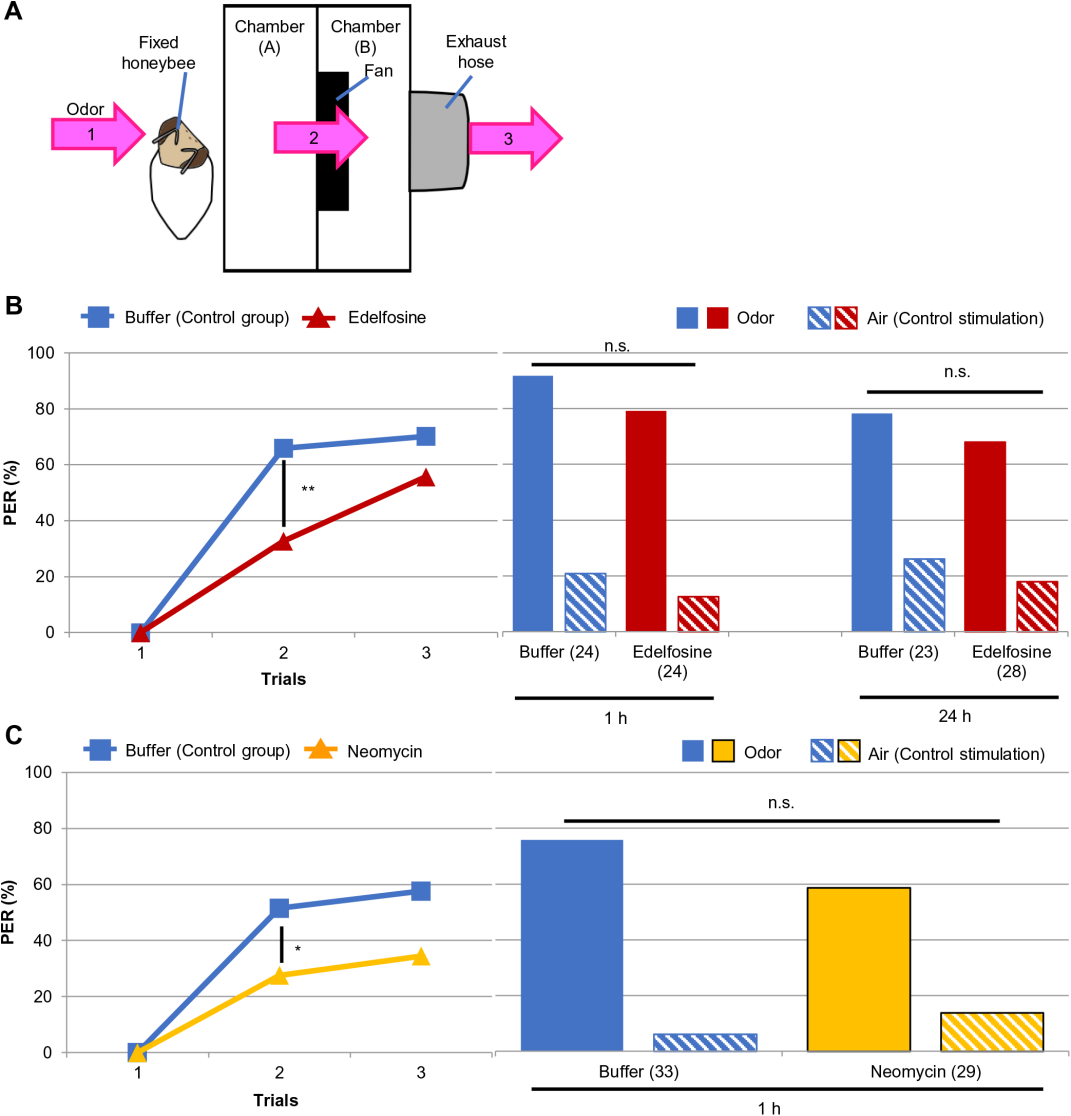
**Effects of edelfosine and neomycin on olfactory associative learning and memory in the honeybee.** (A) Illustration of the experimental setup we used for the behavioral assay. Pink arrows with numbers represent odor stream. The bee received odor stimulation in front of chamber (A) (1). The odor was removed from chamber (A) to chamber (B) and outside by a fan (2) and hose (3). (B,C) Responses of the bees treated with edelfosine (B) or neomycin (C) during learning and memory are shown. Honeybees injected with buffer were used as controls. Left columns represent the bees' responses to odor stimulation in each conditioning trial. All experimental groups exhibited an increased PER with trials (B: control, *P*<1.00×10^−13^; edelfosine, *P*<5.00×10^−10^; C: control, *P*<5.0×10^−8^; neomycin, *P*<0.000 5, Cochran's Q test). Drug-treated groups had a significantly lower response than their controls at the second trial (**P<0.001, *P<0.05, Fisher's exact probability test). Right columns indicate the response of the same bees in the left columns to odor or air stimulation at the 1 h or 24 h memory test. An air puff was used as control stimulation. Response to the odor was significantly higher than that to the air puff in all groups (in B, 1 h: *P*<0.0005 for both groups; 24 h: *P*<0.005 for both groups; in C, control, *P*<5.00×10^−6^; neomycin, *P*<0.005, McNemar's chi-square test). Distribution of the bees responding to odor and air did not differ between treatments in each experiment (*P*>0.05, Mann–Whitney's U test). The numbers of the bees analyzed are represented in parentheses in the right columns. The experiments shown here were performed once. PER, proboscis extension reflex; n.s., not significant.

Based on our findings in the biochemical assay, we used edelfosine and neomycin to examine the effect of PLC inhibition in the olfactory-proboscis extension reflex (PER) associative learning and memory task in the honeybee. We injected edelfosine or neomycin into the head of forager honeybees and trained the animals to learn an association between odor stimulation and a sucrose reward. We presented linalool (46.8% in mineral oil; Szyszka et al., 2008) as the odor by administering it as an air puff from a 50 ml plastic syringe. Three training trials were administered with 10-min inter-trial intervals, and the proportion of responding bees was compared between the drug-treated groups and control groups, which were treated with the injection buffer. Next, we analyzed memory retention at 1 h or 24 h after conditioning using the same experimental groups. Because the odor was presented as an air puff from a plastic syringe in the training, we considered that the bees might have learned an association between sucrose and the air puff, but not the odor. Therefore, we tested memory twice with a 10-min interval, and in each trial bees were presented with the odor or an air puff only, which was used as a control stimulation. We used the experimental setup illustrated in Fig. 5A to present and remove the odor stimulation.

All groups exhibited an increased response to odor stimulation with progression of conditioning (*P*<0.0005 for each group, Cochran's Q test): in the examination of edelfosine, approximately 70% and 56% of control and drug-treated honeybees responded to odor at the last conditioning trial, respectively (Fig. 5B left). When neomycin was investigated, approximately 58% and 34% of control and drug-treated bees exhibited the conditioned response at the third conditioning trial, respectively (Fig. 5C left). These findings suggested that bees in all experimental groups learned the association between the odor and sucrose. When the conditioned response in each trial was compared between treatment groups, however, the drug-treated groups displayed a decreased response to odor stimulation compared with the control groups: edelfosine treatment decreased the conditioned response by approximately 50% and 21% compared with the control group at the second and third sessions, respectively (Fig. 5B left). Neomycin treatment also decreased the number of bees exhibiting a PER to the odor to approximately 54% and 60% compared with control group at the second and third sessions, respectively (Fig. 5C left). Responses of drug-treated groups were significantly different from their controls at the second trial (*P*=0.000879 and 0.0481 for investigation of edelfosine and neomycin, respectively, Fisher's exact probability test). These findings suggested that edelfosine and neomycin treatment disrupted acquisition of the odor-sucrose association.

In the memory test, all groups exhibited a higher response to odor than the control stimulation (*P*<0.005 for each group, McNemar's chi-square test): when edelfosine was investigated, approximately 71% and 67% of control and drug-treated bees displayed odor-specific memory at 1 h, while approximately 57% and 54% of the control and drug-treated bees, respectively, exhibited odor-specific memory at 24 h (Fig. 5B right). In the neomycin experiment, approximately 70% and 48% of the control and drug-treated bees, respectively, displayed odor-specific memory (Fig. 5C right). When the ratio of bees responding to odor and air was compared between treatment groups, no significant difference was detected in any experiment (Fig. 5B and C right). Thus, these findings suggest that drug treatment before training did not affect odor sensing during conditioning and memory retention up to 24 h (edelfosine) or 1 h (neomycin).

We also analyzed the effect of edelfosine and neomycin on mortality and sucrose perception in the honeybee. When mortality was calculated at the end of the training and memory test, no significant difference in mortality between treatment groups was detected in any experiment (Table 2). The proportion of bees without a response to sucrose also did not differ between treatment groups in any experiment, (*P*>0.05, Fisher's exact probability test; Table 3), suggesting that the pharmacologic agents had no detrimental effects on survival or sucrose perception.

**Table 2.**
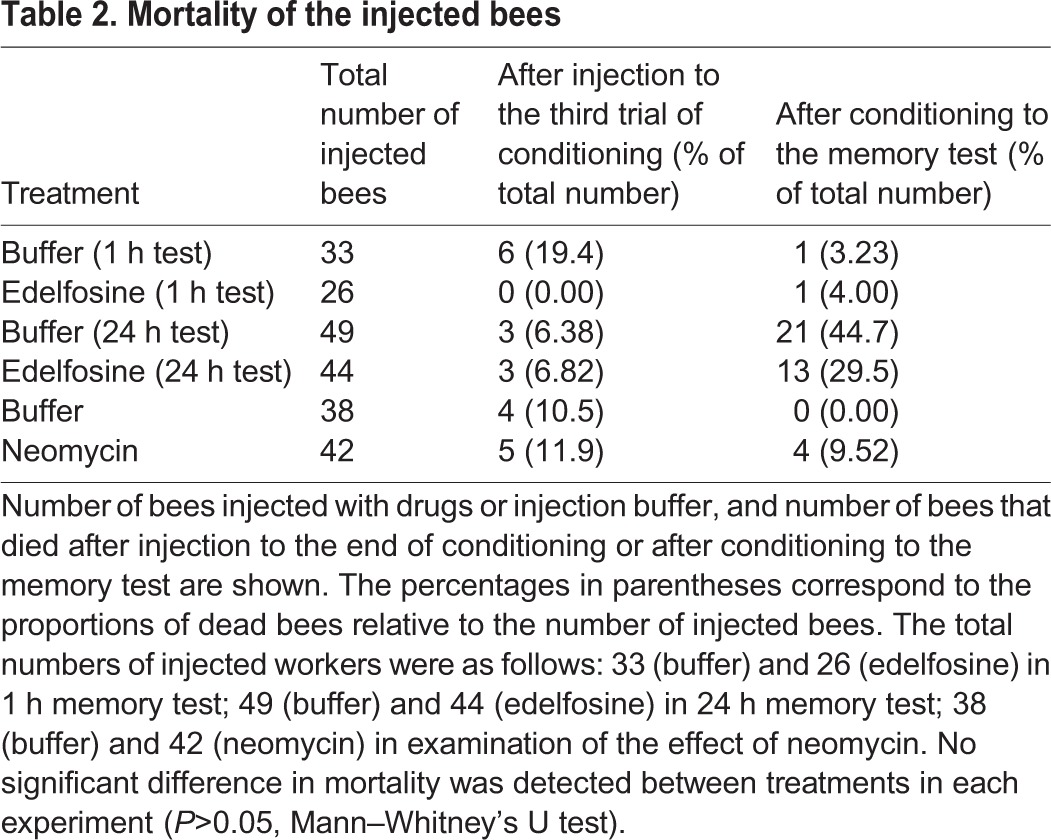
**Mortality of the injected bees**

**Table 3.**
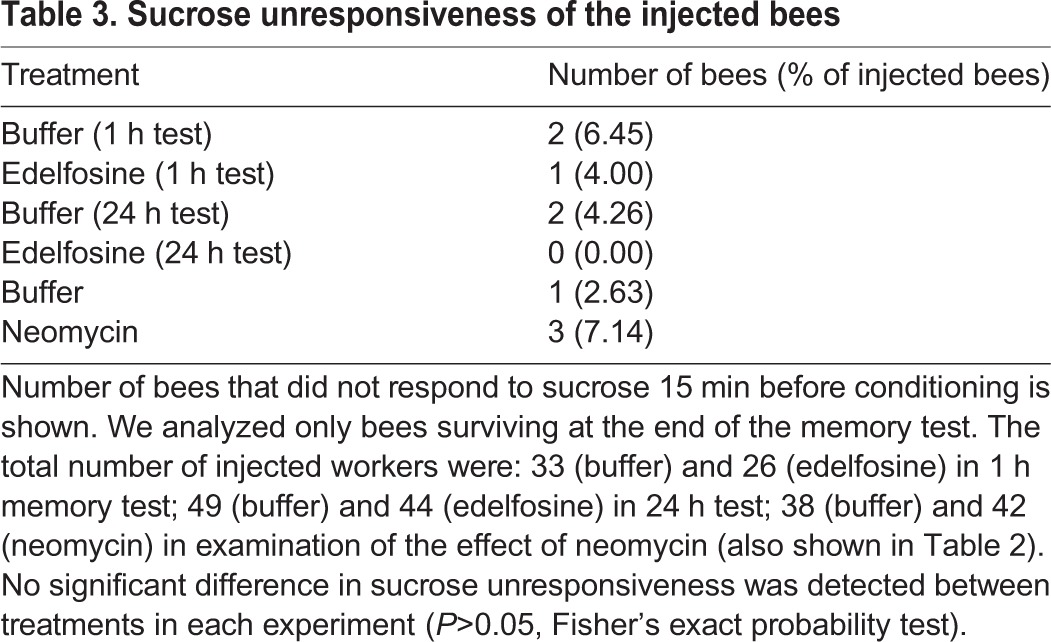
**Sucrose unresponsiveness of the injected bees**

## DISCUSSION

### Overall conservation of PLC subtypes in the honeybee and fruit fly

In mammals, PLC subtypes have different activation mechanisms (Smrcka et al., 2012; Dusaban and Brown, 2015). For example, PLCβ is activated by heterotrimeric G protein downstream of G protein-coupled receptor (GPCR) while PLCγ is activated by small molecule G protein downstream of the receptor coupled with tyrosine kinase (Smrcka et al., 2012). PLCε is unique in that it interacts with both heterotrimeric and small G protein (Smrcka et al., 2012). Therefore, different PLC subtypes are involved in distinct signaling pathways.

Previous studies of gene expression profiles in the honeybee brain identified some PLC subtypes. For example, our previous study demonstrated preferential expression of *PLCe* in the MBs (Suenami et al., 2016). A transcriptomic study also identified the expression of another PLC subtype in the honeybee brain (Sen Sarma et al., 2009). *D*. *melanogaster* has three PLC subtypes, but not PLCe (Greenberg et al., 2012; Balakrishnan et al., 2015). These findings suggest that the profiles of PLC subtypes differ among insect species.

Here, using a blastp search, we identified AmPlc, AmnorpA2, and Amsl as homologues to the fruit fly Plc21C, norpA, and sl, respectively. They showed generally high identity, suggesting functional conservation of these proteins between the honeybee and *D*. *melanogaster*. Because blastp searches using other PLC subtypes as queries in mouse did not detect additional PLC in the honeybee, the profile of PLC subtypes in the honeybee is nearly the same as that in the fruit fly, except for PLCe. This suggests that molecular signaling involving PLCe is unique to the honeybee, while the three other PLC subtypes contribute to signaling shared between honeybee and the fruit fly.

### MB-selective expression of PLC genes in the honeybee

Our findings demonstrated that *PLCe*, *AmPlc*, and *AmnorpA2* are more strongly expressed in the MBs than in the other tissues analyzed. The preferential expression of *PLCe* in the MBs compared with the other brain regions (approximately 19-fold higher, when *RpL32* was used for normalization) was consistent with our previous analysis (Suenami et al., 2016). On the other hand, the relative expression of *AmPlc* and *AmnorpA2*, which was normalized by that of *RpL32*, in the MBs was higher than that in the other brain regions by only approximately 1.7- and 1.9-fold, respectively. Thus, even though the three PLC genes are expressed in the MBs more strongly than the other brain regions, preferential expression in the MBs is unique to *PLCe*, suggesting that PLCe is involved in unique function in the MBs compared with the other PLC subtypes.

We also detected the expression of PLC genes in the retinae, proboscises, and antennae. Previously, *norpA* was reported to be expressed in the fruit fly antennae and labellum (Koganezawa and Shimada, 2002), and a genetic study using RNA interference identified the involvement of *Plc21C* in the gustatory response in the fruit fly (Kain et al., 2010). Electrophysiology in labellum taste receptor cells of the fleshfly detected a response to taste stimulation through PLC (Koganezawa and Shimada, 2002). Furthermore, a homologue of *Plc21C* is expressed in olfactory hairs in the moth antenna (Chouquet et al., 2010). Considering our finding and these reports, expression of PLCβ genes in olfactory and gustatory sensory organs and the function of PLCβ in olfactory and gustatory perception is common among insects.

### Downregulation of PLC activity in brain tissues by edelfosine and neomycin

We found that PLC activity in the MB homogenate was lower than that in the other brain regions. This was unexpected based on the results of the qRT-PCR experiment. The relative expression of PLC genes was always higher in the MBs than in the other brain regions although we used *RpL32*, *EF1a-F2*, and *Arp1* as reference genes (Fig. 3; Fig. S2), indicating that comparison of relative expression levels between brain regions in qRT-PCR was not problematic. Rather, we speculate that the translation rate differed between brain regions, leading to a difference in the amount of PLC proteins. Moreover, because mammalian PLCβ and PLCε are located at the plasma membrane (Lopez et al., 2001) and we did not separate membrane and cytoplasmic fractions in the brain homogenates, it is also possible that a proportion of the membrane fraction was higher in the homogenate from the MBs than the other brain regions, and thus the concentration of free PLC was lower in the MBs. These possibilities should be examined by quantification and comparison of total or membrane-bound PLC proteins between these brain regions.

We used edelfosine and neomycin as potential PLC inhibitors and detected a decrease in PLC activity in the reaction mixtures containing these inhibitors. Although it is not known how these drugs inhibit PLC, edelfosine, which is derived from ether, is thought to be integrated into the plasma membrane where it affects PLC activity (Powis et al., 1992). Neomycin is an aminoglycoside and thought to competitively inhibit PLC based on the study of another aminoglycoside (Lipsky and Lietman, 1982). Neomycin is also considered to bind PLC substrates and affect PLC activity (Lipsky and Lietman, 1982). Thus, these drugs are thought to inhibit PLC through different mechanisms. Because edelfosine and neomycin share only PLC as target, and both drugs decreased PLC activity, we concluded that they can be used as PLC inhibitors in the honeybee brain. We did not discriminate the activity of distinct PLC subtypes in the present study, as the specificity of PLCglow and the PLC inhibitors for each PLC subtype is unknown.

### Involvement of PLC in acquisition but not in memory retention for up to 24 h

Here, we identified the involvement of PLC in learning and memory in an insect for the first time. Both edelfosine and neomycin decreased PER to odor stimulation in the conditioning trials, while no detrimental effect on survival or sucrose perception was detected. Because these drugs are suggested to inhibit PLC through different mechanisms and share only PLC as a common target (see above), our results strongly suggest that the attenuated learning in bees treated with these inhibitors was due to the decrease in PLC activity and that PLC enhances task acquisition in the honeybee. In contrast to the attenuated task acquisition by drug injection, no difference in memory retention was detected between treatments at 1 h and 24 h after conditioning. This finding suggested that PLC activity in conditioning is not related to memory retention for up to 24 h.

The animal's response during task acquisition comprises detection of the association between the odor and reward, consolidation, retention, and recall of memory (Abel and Lattal, 2001). As we did not discriminate these processes in the present study, the process in task acquisition that involves PLC in the honeybee remains unclear. Moreover, the contribution of PLC to memory formation after task acquisition was not examined as we injected PLC inhibitors only before, and not after, training. To investigate these points, it will be useful to examine the involvement of PLC in consolidation, retention and recall by injecting PLC inhibitors immediately after conditioning (for consolidation and retention) and immediately before memory test (for recall).

### Possible intracellular signaling through which PLC regulates honeybee olfactory appetitive learning

In the present study, PLC inhibitors did not completely block task acquisition. Because we did not inject PLC inhibitors into a specific brain region, the inhibition of PLC was insufficient in the brain structure that is required for learning in the honeybee. Multiple molecular pathways and/or neural mechanisms are involved in learning. For example, both the ALs and MBs are involved in learning (Hammer and Menzel, 1998; Lozano et al., 2001). Furthermore, the MBs also utilize a molecular pathway without PLC in learning. When *NR1*, which encodes a subunit of the N-methyl-D-aspartate receptor, is knocked down in honeybee MBs, learning is impaired (Müßig et al., 2010). These pathways may have prevented the complete loss of task acquisition in the present study.

Then, what are the proteins functioning upstream and downstream of PLC in learning in the honeybee? A GPCR is likely to activate PLC in honeybee learning. This is because GPCR activates PLCβ and PLCε in mammals and we identified PLCe, AmPlc, and AmnorpA2, which are honeybee homologues of PLCβ or PLCε, as candidates involved in task acquisition in this study. Previous studies using pharmacology or RNA inhibition identified two GPCRs involved in learning in the honeybee: the octopamine receptor and muscarinic acetylcholine receptor (mAChR) (Farooqui et al., 2003; Lozano et al., 2001). The octopamine receptor increases intracellular calcium when heterologously expressed in a HEK 293 cell line (Grohmann et al., 2003; Beggs et al., 2011), and thus PLCβ was suggested to be activated by octopamine receptor activity (Beggs et al., 2011). Our finding that PLC is involved in learning in which octopamine receptors are also involved, is consistent with this suggestion. mAChR is involved in recall of olfactory appetitive memory (Lozano et al., 2001). Although the relation between PLC and mAChR in the honeybee has not been revealed, PLC is activated downstream of mAChR in the grasshopper brain (Wenzel et al., 2002). Thus, this receptor might activate PLC in learning in the honeybee if PLC is involved in memory recall. Although receptor tyrosine kinases, which can activate mammalian PLCε (Smrcka et al., 2012; Dusaban and Brown, 2015), are other candidate molecules for activating PLCe in honeybee learning, there are no studies examining the involvement of this type of receptor in learning in the honeybee.

Based on current knowledge of calcium signaling, PLC opens IP_3_Rs by generating IP_3_, leading to an increase in the intracellular calcium concentration and activation of PKC and CaMKII (Smrcka et al., 2012; Dusaban and Brown, 2015). This implicates these factors as possible targets of PLC in appetitive learning and memory in the honeybee. In fact, long-term memory formation is attenuated when an intracellular calcium increase is blocked by a chelator (Perisse et al., 2009) and CaMKII is inhibited either pharmacologically or by small interfering RNA during training (Matsumoto et al., 2014; Scholl et al., 2015). Thus, sufficient activation of PLC in conditioning might trigger long-term memory formation through an increase in intracellular calcium and CaMKII activation. On the other hand, given that inhibiting an increase in the calcium concentration or CaMKII during conditioning does not affect learning (Perisse et al., 2009; Matsumoto et al., 2014; Scholl et al., 2015), calcium signaling might not be related to learning. We speculate that small G proteins might be involved downstream of PLC in learning, because small G proteins interact with PLCε in mammalian cell lines (Smrcka et al., 2012; Dusaban and Brown, 2015) and are suggested to be involved in olfactory learning and memory in *D. melanogaster* (Moressis et al., 2009), though involvement of small G proteins in honeybee appetitive learning has not been tested.

PLC is involved in learning and memory in mammals. For example, knockout of *PLCβ4* in the mouse impairs associative eye-blink learning (Miyata et al., 2001). A mutation in *PLCε* that eliminates the ability to generate IP_3_ improves learning (Quan et al., 2012). Injecting the PLC inhibitor U73122 into the mouse premotor cortex impairs motor skill acquisition (Rioult-Pedotti et al., 2015). Generally, disruption of PLC in these studies commonly changed learning in the mouse. These and our studies are similar in that PLC inhibition affected acquisition. More studies are required to examine whether mammals and honeybees share similar molecular mechanisms in learning through PLC.

In contrast to studies using mammals, there are few studies on the function of PLC in learning and memory in insects. A study comprehensively searching for genes involved in olfactory aversive learning and memory in *D. melanogaster* detected *Plc21C* and *norpA* (Walkinshaw et al., 2015). The detected changes in the fruit flies in which these genes were knocked down, however, might not be due to impaired learning and memory ability, because these animals also exhibited other physical or behavioral abnormalities (Walkinshaw et al., 2015). The contribution of PLC to learning and memory in insects therefore was unknown before the present study. Is the involvement of PLC in appetitive learning prevalent among insects? Some research suggests species-specific differences in molecular mechanisms underlying insect learning and memory (Mizunami et al., 2015). For example, although a mutation in *rutabaga*-adenylate cyclase attenuates learning in *D*. *melanogaster* (McGuire et al., 2005; Trannoy et al., 2011), pharmacologic inhibition of adenylate cyclase does not affect learning in cricket or honeybee (Matsumoto et al., 2006; Matsumoto et al., 2014). PLC might also contribute to differences between honeybee and *D*. *melanogaster* if PLCe is involved in acquisition in honeybee. We did not discriminate the contribution of each PLC subtype to task acquisition in the present study, however, as we used a pharmacologic approach. To clarify this point, application of the previously reported genome editing method in the honeybee (Kohno et al., 2016) will be useful.

### Concluding remarks

In the present study, we showed that *PLCe* and two genes encoding PLCβ are expressed more strongly in the MBs than in the other brain regions or peripheral sensory organs in the honeybee. We also demonstrated for the first time that pharmacologic PLC inhibition attenuated task acquisition, but not memory retention up to 24 h, suggesting that PLC is involved in olfactory appetitive learning in the honeybee. The results presented here enhance our understanding of species-specific molecular mechanisms of learning and memory in insects.

## MATERIALS AND METHODS

### Animal

The colony of European honeybees (*A*. *mellifera* L.) was purchased from local distributors and maintained at the University of Tokyo. Foragers (female bees) with pollen bags on their hind legs, whose ages were unknown, were caught at the hive entrance and used in all analyses except for the identification of PLC subtypes using bioinformatics.

### Identification of PLC subtypes coded in the honeybee genome

A blastp search was performed of the NCBI database using amino acid sequences of PLC subtypes (Plc21C, norpA, and sl) from the fruit fly (*D*. *melanogaster*) as queries. We also performed a blastp search using PLCδ, PLCη, and PLCζ in the mouse (*M*. *musculus*) as queries.

### Phylogenetic tree construction

Amino acid sequence data of PLC subtypes in the honeybee, fruit fly, mouse, human (*H*. *sapiens*), and yeast (*S*. *cerevisiae*) was obtained from the NCBI database. After ClustalW alignment using these sequences, a phylogenetic tree was constructed by the neighbor-joining method. The following PLC subtypes were used (accession numbers are given in parentheses): honeybee AmPlc, AmnorpA2, Amsl, and PLCe (XP_003250991.1, XP_016769634.1, XP_016771738.1, and XP_016767302.1); *D*. *melanogaster* Plc21C, norpA, and sl (NP_995605.1, NP_001162661.1, and NP_476726.2); mouse PLCβ1, PLCβ2, PLCβ3, PLCβ4, PLCγ1, PLCγ2, PLCδ1, PLCδ4, PLCε1, PLCζ1, PLCη1, and PLCη2 (NP_001139302.1, NP_001277719.1, NP_001277278.1, NP_038857.1, NP_067255.2, NP_758489.1, NP_001280577.1, NP_683739.2, NP_062534.2, NP_473407.2, NP_899014.2, and NP_001106831.1); human PLCβ1, PLCβ2, PLCβ3, PLCβ4, PLCγ1, PLCγ2, PLCδ1, PLCδ3, PLCδ4, PLCε1, PLCζ1, PLCη1, and PLCη2 (NP_056007.1, NP_004564.2, NP_000923.1, NP_001166117.1, NP_002651.2, NP_002652.2, NP_001124436.1, NP_588614.1, NP_116115.1, NP_057425.3, NP_149114.2, NP_001124432.1, and NP_055453.2); and yeast PLC1 (NP_015055.1). If a gene had variants, amino acid sequence of the longest isoform was used.

ClustalX2 (Larkin et al., 2007; www.clustal.org/clustal2/), treeview (Page, 1996; http://taxonomy.zoology.gla.ac.uk/rod/treeview.html), and BioEdit (www.mbio.ncsu.edu/bioedit/bioedit.html) software were used in the alignment, phylogenetic tree construction, and calculation of identity, respectively.

### qRT-PCR

The MBs, other brain regions, retinae, proboscises, and antennae were dissected from forager bees under a binocular microscope and quickly frozen with dry ice or liquid nitrogen. Dissection of the MBs and other brain regions were performed as previously described (Suenami et al., 2016). Proboscises and antennae were cut into pieces with scalpels and tweezers for easy homogenization. We used two bees as a lot, and a total of five lots was analyzed.

Tissues were homogenized in TRIzol LS reagent (Ambion) and total RNA was extracted. Two sets of RNA samples were prepared for analysis of *PLCe* and the other PLC genes. RNA (100 ng for analyzing *PLCe* or 200 ng for analyzing *AmPlc*, *AmnorpA2*, and *Amsl*) was reverse-transcribed with a PrimeScript RT Reagent Kit with gDNA eraser (Perfect Real-Time; TaKaRa, Shiga, Japan). qRT-PCR was performed using SYBR Premix ExTaq II (Tli RNase H Plus; TaKaRa) and LightCycler (Roche). Reverse transcription and qRT-PCR were performed according to manufacturer's instructions. The following gene-specific primers were used in qRT-PCR: *PLCe* (XM_392335.4), 5′-GTTTCGCCAATCGAAAAACG-3′ and 5′-CGAATACCAGCTGTTCTACC-3′; *AmPlc* (XM_006567226.2), 5′-TGCAATCCAAGACAGCAAGC-3′ and 5′-TCAAGAGGAGCATCCAGCAA-3′; *AmnorpA2* (XM_016914145.1), 5′-AATTCTGGGCGATCTGCTTC-3′ and 5′-TTCCAACTCGTGCTTCTCCA-3′; *Amsl* (XM_016916249.1), 5′-AATGGCCAGAAGATGCAAAA-3′ and 5′-TGAACCAAGCGACGTAATCC-3′; *RpL32* (XM_006564315.1), 5′-AAAGAGAAACTGGCGTAAACC-3′ and 5′-CTCGTCATATGTTGCCAACTG-3′; *EF1α-F2* (XM_006569890.1), 5′-TTGGTTTAAGGGATGGACGG-3′ and 5′- TGTGTTGAAACCAGGTATGG-3′; *Arp1* (NM_001185146.1), 5′-TCCCCGAATCCCGAAAG-3′ and 5′-CGGAGGAACCAAAGGACAA-3′. Expression of PLC genes was normalized by *RpL32*. Statistical analysis was performed using EZR software (Kanda, 2013). Kruskal–Wallis test was performed to detect difference of expression levels among all tissues. When significant difference was detected in this test, Steel-Dwass test was also performed to compare expression levels between tissues.

### PLC activity assay

After the foragers were anesthetized on ice for more than 30 min as described previously (Kamikouchi et al., 2000), the MBs and other brain regions were dissected under a binocular microscope as described above and quickly frozen with liquid nitrogen. We used two bees as a lot, and six lots were prepared.

Tissues were homogenized in 50 mmol/l HEPES-KOH (pH 7.2), 70 mmol/l KCl, and 1.0 mmol/l CaCl_2_. These tissues were centrifuged for 10 min at 4000 rpm (approximately 900 ***g***) and 4°C, and the supernatant was centrifuged again for 20 min at 13,000 rpm (approximately 9500 ***g***) and 4°C as described previously (Yoshioka et al., 1985). The supernatant was obtained and stored at −20°C. Protein concentrations in the homogenate were measured using Pierce BCA Protein Assay Kit (ThermoFisher Scientific) (Zhu et al., 1993) and Pierce Bovine Serum Albumin Standard Ampules 2 mg/ml (ThermoFisher Scientific).

To detect PLC activity, we used PLCglow (KXT Bio), a fluorogenic substrate (Huang et al., 2011), and the reaction was performed according to the manufacturer's instruction. The reaction mixture contained 12.5 µmol/l PLCglow, 50 mmol/l HEPES-KOH (pH 7.2), 70 mmol/l KCl, 1.0 mmol/l CaCl_2_, 2.0 mmol/l dithiothreitol, 50 mg/l bovine serum albumin, and homogenate containing 1.3 μg protein. The final volume of reaction mixture was 10 μl. The reaction mixture was incubated for 30 min at 25°C in a thermal cycler and 2 μl of 25 mmol/l ethylene glycol bis (β-aminoethylether)-N, N, N′, N′-tetraacetic acid was added to stop the reaction. The reaction mix was centrifuged for 5 min at 2000 rpm (approximately 310 ***g***), and 10 μl of supernatant was applied to a black, low volume 384-well round-bottom microplate (Corning). Fluorescence was quantified using a Gemini EM microplate reader (Molecular Devices) at excitation and emission wavelengths of 344 nm and 530 nm, respectively, and automix was performed for 5 s before quantification. Quantification was performed three times as a technical triplicate and mean fluorescence was used for the analyses. To remove the fluorescence from the homogenate or intact substances from statistical analysis, we also analyzed two kinds of control mixes: one containing only homogenate and the other containing only substrate. The difference in the fluorescence signal between the reaction mix and control mixes was statistically analyzed.

To examine the inhibitory effects of edelfosine and neomycin on PLC, these drugs were added to the reaction mix at the following final concentration: edelfosine, 1.0 mmol/l; neomycin, 0.55 mmol/l. Both inhibitors were purchased from Sana Cruz Biotechnology, Inc. According to the manufacturer's instructions, stock solutions were made with sterilized water at the following concentrations: edelfosine, 5.0 mmol/l; neomycin, 550 mmol/l. Stock solutions were stored at −20°C (edelfosine) or 4°C (neomycin). Here, we constructed additional mixes to remove the fluorescence from reactions other than interactions among the homogenates, substrates, and drugs: one containing substrate and inhibitor, another containing inhibitor and homogenate, and the other containing only inhibitor. Fluorescence detected in the former two mixes was subtracted, and that derived from the last mix was added to the fluorescence in the reaction mix containing all of homogenates, substrates, and drugs.

EZR software (Kanda, 2013) was used for the statistical analysis.

### Odor-PER associative learning and memory

The experimental system used was as described previously (Hori et al., 2006; Felsenberg et al., 2011; Matsumoto et al., 2014; Scholl et al., 2015). Experiments were performed from the end of May to the end of June in 2016 and from the end of March to the end of April in 2017 for edelfosine, and in September for neomycin in 2016.

Foragers were randomly caught in the afternoon to evening and anesthetized on ice. The bee was harnessed in a 0.5 ml plastic tube. To expose only the bee's head, parafilm was used. A small amount of cotton was placed at the bottom of the tube to support the bee's body. We placed the tube into a 1.5 ml plastic tube on a tube rack, which allowed us to handle the animals easily. The bee was kept at 27°C until manipulation. In the evening, 0.88 mol/l sucrose dissolved in tap water was fed to the bee until satiation [a 4 μl (0.88 mol/l sucrose) drop was repeatedly presented to the bee until the PER stopped]. The bee was then placed back at 27°C.

Drug treatment and olfactory conditioning were performed the next day. Drug application was started 90 min before conditioning. After the bee was immobilized on ice for 3 min, a small hole was opened at the medial ocellus using an insect pin. A fine injection needle was inserted into the bee's head through the hole. The injection needle was made of borosilicate glass capillary (Sutter Instrument) as follows: after the capillary was pulled, the tip was manually cut to ∼20 μm under a binocular microscope. Approximately 330 nl of 1.0 mmol/l edelfosine, 450 mmol/l neomycin, or control solution was injected. Edelfosine or neomycin stock solution was dissolved in injection buffer with following composition: 10 mmol/l HEPES-NaOH (pH 6.7), 130 mmol/l NaCl, 6.0 mmol/l KCl, 4.0 mmol/l MgCl_2_, 5.0 mmol/l CaCl_2_, 25 mmol/l glucose, and 0.16 mol/l sucrose (Fiala et al., 1999). This injection buffer was also used as control solution. Bees with hemolymph flowing out from the holes were discarded from the analysis. The remaining bees were maintained at 27°C until conditioning. We injected buffer and drug to nearly half of workers in each experimental session, and the order of injection was changed among sessions. This was to equalize the effect of time after injection on action of the injected solutions between treatment groups. It was also expected to equalize the effect of experience of injection on worker's behavioral performance between treatment groups.

The bee was next placed beside the experimental setup (Fig. 5A) and familiarized to the setup for 30 min before conditioning. After 15 min, we moved the bee to another room and presented it with 1.25 mol/l sucrose solution (dissolved in tap water) to examine the normal sucrose perception of the bee. To do this, the antennae of the bee were touched with a micropipette containing the sucrose solution and we recorded whether the bee showed a PER. Bees not showing a PER were discarded (Scholl et al., 2015).

The conditioning paradigm consisted of three trials. In each trial, the bee was placed in front of the chamber (A) (Fig. 5A) and familiarized with the setup for 10 s. Linalool dissolved in mineral oil at 46.8% (Szyszka et al., 2008) was presented as an odor stimulation for 5 s. To do this, a 4 μl droplet of diluted linalool was placed on a filter paper, and this paper was set in a 50 ml plastic syringe. A 20 ml air puff containing the odor was presented to the bee by manually pushing the syringe. After 3 s of odor presentation, 1.25 mol/l sucrose solution as reward was presented for 4 s. The reward was presented by touching the antennae with a toothpick immersed in sucrose solution. When the bee exhibited a PER after sucrose presentation, the bee was fed the reward. The bee was allowed to rest for 13 s after the sucrose presentation and then returned to the tube rack. An association between odor and sucrose was expected to occur in the 2 s when the presentation of odor and sucrose overlapped. Bees displaying a PER to odor in the second/third trial were considered to have learned the association between odor and sucrose. The number of bees exhibiting such responses was statistically analyzed. The intertrial interval was 10 min. After conditioning, the bee was allowed to rest at 27°C until the memory test. Bees whose 24 h memory was tested were fed in the evening, as described previously.

The memory test was performed 1 h or 24 h after conditioning. The test consisted of two trials with a 10 min interval. The bee was placed next to the experimental setup for 30 min before the test. In each trial, after 10 s of familiarization to the setup in front of the chamber (A) (Fig. 5A), the bee was presented with an odor or an air puff for 5 s. The air puff was used as a control stimulation. Then, the bee was allowed to rest for 15 s and returned to the tube rack. The order of odor and air stimulation in the first and second trial was changed for half of the bees in each treatment group to minimize the effect of the experience in the first trial on performance in the second trial. Bees exhibiting a PER to the odor or air stimulation were considered to have retained memory of the odor or air, respectively, and the number of bees was statistically analyzed. After the memory test, the bee's sucrose perception was again examined as previously described and bees not exhibiting PER to sucrose were discarded (Matsumoto et al., 2014).

Mortality after drug application and lack of response to sucrose were also statistically analyzed. The numbers of workers used in the drug application experiments were as follows: 33 (control) and 26 (edelfosine) in examination of the effect of edelfosine on 1 h memory formation; 49 (control) and 44 (edelfosine) in the examination of the effect of edelfosine in 24 h memory test; 38 (control) and 42 (neomycin) in the examination of the effect of neomycin (also see Table 2). The numbers of workers discarded in the behavioral analyses are shown in Tables 2 and 3. The numbers of workers whose learning and memory was statistically analyzed were as follows: 24 (control and edelfosine) in examination of 1 h memory formation; 23 (control) and 28 (edelfosine) in examination of 24 h memory test; 33 (control) and 29 (neomycin) in examination of neomycin treatment (also see Fig. 5).

Statistical analysis was performed using the Statcel 4 program (Yanai, 2015). We used Cochran's Q test to detect significant increase in the numbers of workers which learned association between odor and sucrose in each treatment group. Fisher's exact probability test was performed to examine significant difference in associative learning between experimental groups in the second and third training trial. Retention of odor memory was examined by McNemar's chi-square test in each experimental group. Then, response to odor and air puff in memory test was compared between experimental groups. Sucrose unresponsiveness was analyzed by Fisher's exact probability test between experimental groups. For statistical test of mortality, see Table 2.

## Supplementary Material

Supplementary information
