# Supplementary material for: Pharmacologic inhibition of phospholipase C in the brain attenuates early memory formation in the honeybee (Apis mellifera L.)
Source: Biol Open. 2018 Jan 15;7(1):bio028191. doi: 10.1242/bio.028191 (PMC5829494; doi:10.1242/bio.028191)
Supplement: Supplementary information [file biolopen-7-028191-s1.pdf]

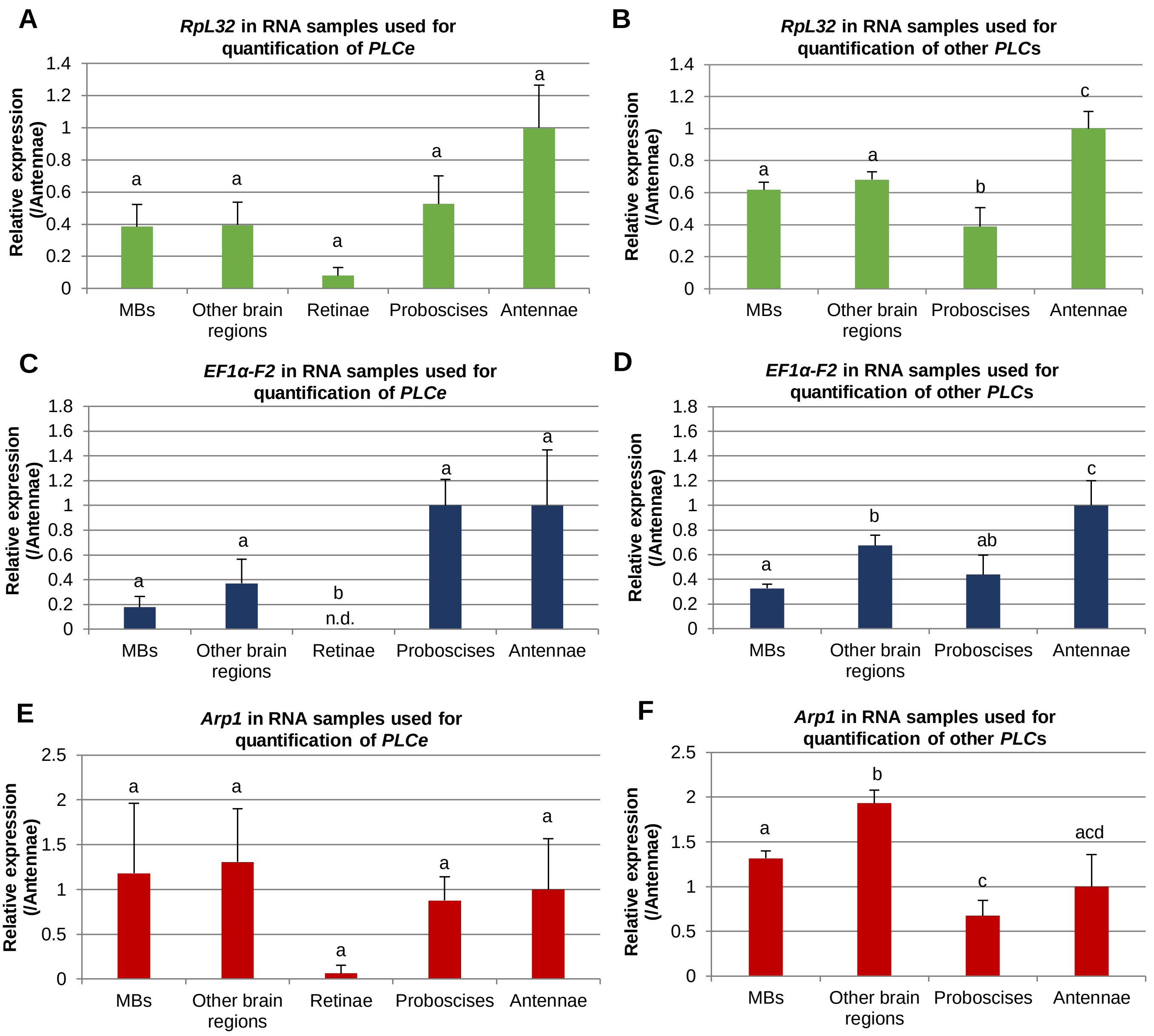


**Supplementary Fig. S1. qRT-PCR analysis of *RpL32*, *EF1α-F2*, and *Arp1* in tissues involved in learning and memory in the honeybee**

*RpL32* (A and B), *EF1α-F2* (C and D), and *Arp1* (E and F) in RNA samples of the MBs, other brain regions, retinae, proboscises, and antennae were quantified by qRT-PCR. Because we analyzed *PLCe* and the other PLC genes using different RNA samples, expression of the above three genes in these RNA samples was analyzed separately. Relative expression levels, taking expression in the antennae as one, are shown in each panel. Bars and lines indicate mean of expression levels and s.d. of five lots of RNA samples, respectively. A significant difference in expression levels was detected in all analyses (A and B: $P < 0.001$; C: $P < 0.000\,5$; D: $P < 0.001$; E: $P < 0.05$; F: $P < 0.005$, Kruskal-Wallis test). Different letters on the bars represent a significant difference ($P < 0.05$, Steel-Dwass test). Expression of *EF1α-F2* was not detected in any retina samples, while expression of *Arp1* was not detected in three of the five retina samples. n.d., not detected; s.d., standard deviation. *Arp1*, *actin related protein 1*; *EF1α-F2*, *elongation factor 1 alpha-F2*; *PLCe*, *phospholipase C epsilon*; *RpL32*, *Ribosomal protein L32*.

Biology Open • Supplementary information

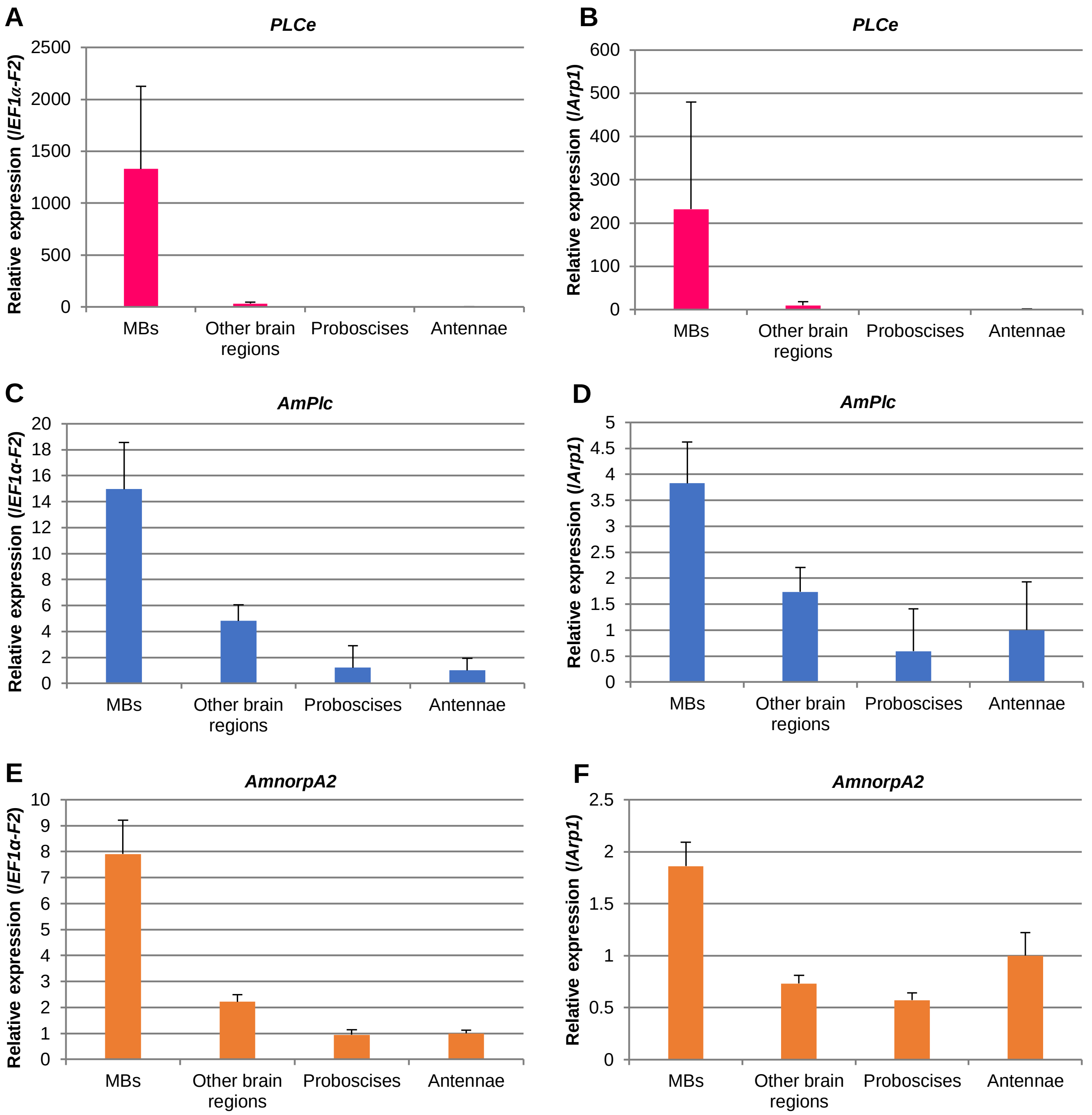


**Supplementary Fig. S2. Expression levels of *PLCe*, *AmPlc*, and *AmnorpA2* relative to *EF1α-F2* and *Arp1***
Relative expression of *PLCe* (A and B), *AmPlc* (C and D), and *AmnorpA2* (E and F) in RNA samples of the MBs, other brain regions, proboscises, and antennae are shown in each panel. Expression of the PLC genes was normalized by *EF1α-F2* (A, C, and E) or *Arp1* (B, D, and F), respectively, taking expression in the antennae as one. Bars and lines indicate mean of expression levels and s.d. of five lots of RNA samples, respectively. Expression of *PLCe* in the retinae was not analyzed because expression of *EF1α-F2* was not detected in any retinae samples, and *Arp1* was detected in only two RNA samples. s.d., standard deviation. *AmPlc*, *Apis mellifera phospholipase C*; *AmnorpA2*, *Apis mellifera no receptor potential A2*; *Arp1*, *actin related protein 1*; *EF1α-F2*, *elongation factor 1 alpha-F2*; *PLCe*, *phospholipase C epsilon*.

Biology Open • Supplementary information
